# Aqueous humor and serum 25-Hydroxyvitamin D levels in patients with cataracts

**DOI:** 10.1186/s12886-019-1293-9

**Published:** 2020-01-06

**Authors:** Min-Chul Cho, Rock-Bum Kim, Ja-Young Ahn, Woong-Sun Yoo, Seong-Jae Kim

**Affiliations:** 1 Department of Laboratory Medicine, Gyeongsang National University Hospital and Gyeongsang National University College of Medicine, Jinju, South Korea; 2 Institute of Health Science, Gyeongsang National University, Jinju, South Korea; 3 Department of Preventive Medicine, Gyeongsang National University Hospital and Gyeongsang National University College of Medicine, Jinju, South Korea; 4 Department of Ophthalmology, Gyeongsang National University Hospital and Gyeongsang National University College of Medicine, 79 Gangnam-ro, Jinju-si, Gyeongsangnam-do 52727 South Korea

**Keywords:** 25-hydroxyvitamin D, Aqueous humor, Cataract

## Abstract

**Background:**

Serum 25-hydroxyvitamin D (25 (OH) D) levels are associated with various pathologic ocular conditions. Few studies have assessed 25 (OH) D concentrations in non-serum specimens, and none to date has assessed 25 (OH) D concentrations in human aqueous humor and their association with ocular diseases. This study investigated the possible correlations between 25 (OH) D concentrations in aqueous humor and serum and whether vitamin D concentrations in aqueous humor were associated with cataract.

**Methods:**

This study prospectively enrolled 136 patients, including 87 with senile cataract and 49 with diabetic cataract, who underwent cataract surgery from January to November 2017. 25 (OH) D was measured in aqueous humor and serum specimens collected from all patients, and their correlation was analyzed statistically. Clinical and laboratory data, including the results of ophthalmologic examinations, were compared in the two groups of cataract patients.

**Results:**

No correlation was observed between 25 (OH) D concentrations in aqueous humor and serum (*P* = 0.381). 25 (OH) D concentrations in aqueous humor were significantly higher in patients with diabetic than senile cataract (*P* = 0.006). Multivariate logistic regression analysis showed that the adjusted odds ratio for diabetic cataract for the highest compared with the lowest quartile of 25 (OH) D concentration in aqueous humor was 4.36 ng/ml (95% confidence interval [CI]: 1.33–14.34 ng/ml; *P* = 0.015). Multivariate linear regression analysis showed that 25(OH) D concentration in aqueous humor was 2.68 ng/ml (95% CI: 0.34–5.01 ng/ml; *P* = 0.025) higher in patients with diabetic than senile cataract.

**Conclusions:**

25(OH) D concentrations in aqueous humor and serum did not correlate with each other. Higher 25(OH) D level in aqueous humor was associated with diabetic cataract. These findings suggest that studies of vitamin D levels in patients with ocular conditions should include measurements of vitamin D levels in aqueous humor.

## Background

Vitamin D is a multifunctional molecule that plays significant roles in various biological functions, including the regulation of calcium homeostasis [1]. For example, vitamin D has been shown to reduce inflammatory mediators and have anti-oxidative activities [2]. Many studies show an inverse relationship between vitamin D concentrations and chronic diseases associated with chronic inflammation, including diabetes mellitus, hypertension, heart disease, multiple sclerosis, schizophrenia, and rheumatoid arthritis [3–6].

Two forms of vitamin D have been identified: D2 (ergocalciferol) and D3 (cholecalciferol). Vitamin D status is determined primarily by measuring serum concentration of total 25-hydroxyvitamin D (25 (OH) D), which includes both forms of vitamin D [7]. Although there is no consensus regarding optimal concentrations of total 25(OH) D, individuals with serum concentrations under 20 ng/mL (50 nmol/L) are generally considered vitamin D deficient and those with serum concentrations of 20–30 ng/mL (50–75 nmol/L) are generally considered vitamin D insufficient [8–10]. LC-MS/MS is generally considered the gold standard for the measurement of 25(OH) D [11], but this method has several limitations preventing its use in general clinical laboratories. Thus, 25(OH) D is usually measured by automated equipment using a chemiluminescence-based method, which has shown good correlation with LC-MS/MS [11, 12].

Serum (25 (OH) D) status may also be associated with pathologic ocular conditions. For example, serum vitamin D levels are reported to influence the development of ocular pathologies, such as myopia, age-related macular degeneration, diabetic retinopathy, uveitis, and dry eye syndrome [13–16]. Few studies, however, have investigated vitamin D levels in non-serum specimens such as tears [17–19], and none to date has directly measured vitamin D levels in human aqueous humor specimens.

Aqueous humor is a clear liquid that occupies the anterior segments of the eye. Although aqueous humor derives from blood plasma, its composition differs from serum, with aqueous humor having lower concentrations of protein and glucose and higher concentrations of ascorbic acid than serum. Aqueous humor provides oxygen and nutrients to ocular tissues and removes their waste products. Because the lens is an avascular internal ocular organ supplied by the aqueous humor with oxygen and nutrients, changes in the composition of aqueous humor can lead to cataracts. Studies have reported that changes in several factors in the anterior chamber of the eye are associated with cataract development [20–23].

Cataract is a sight-threatening ocular disease, responsible for more than 30% of all cases of blindness [24, 25]. Factors that increase the risk of cataract include aging, diabetes mellitus, eye trauma, exposure to ultraviolet light, drug use, and other ocular diseases [26–28]. Several epidemiological studies suggest that the serum concentration of vitamin D is associated with the development of age-related or diabetic cataract [16, 29]. To date, however, studies have only examined the association between serum vitamin D levels and ocular diseases, with no studies to our knowledge assessing the range of 25(OH) D concentrations in human aqueous humor or the association between vitamin D concentrations in aqueous humor and cataract. This study therefore measured 25(OH) D concentrations in aqueous humor and serum samples from 136 patients with cataract, including 87 with senile cataract and 49 with diabetic cataract. The primary objective was to determine the correlation between 25(OH) D concentrations in aqueous humor and serum. This study also analyzed the association of vitamin D concentrations in aqueous humor with cataract.

## Methods

### Study design

This was a prospective study conducted at Gyeongsang National University Hospital, Jinju, Korea. Aqueous humor and serum samples were collected from 136 patients who underwent cataract surgery between January and November 2017. The study was approved by the Gyeongsang National University Hospital Institutional Review Board (2017–01-011). The study protocol confirmed to the tenets of the Declaration of Helsinki, and all patients provided written informed consent before participating.

### Ophthalmic examinations and cataract surgery with anterior chamber paracentesis

After a complete medical history was taken, all participants underwent an ophthalmologic examination, including measurements of best-corrected visual acuity and intraocular pressure, as well as slit lamp biomicroscopy, and fundus examination. The enrolled patients were divided into those with senile and diabetic cataracts. Patients with complicated cataracts, including those with cataracts due to eye trauma, uveitis, or long-term use of steroids, and those who had undergone previous ophthalmic surgery were excluded. The diabetic cataract group included patients with well-controlled diabetes (hemoglobin A1C < 7%) with non-proliferative diabetic retinopathy; patients with proliferative diabetic retinopathy and those who had undergone vitrectomy or intravitreal injection were excluded. Patients with diabetes and other ocular diseases (e.g., glaucoma or retinal disease) were excluded from the senile cataract group. Cataract type and severity were graded using the Lens Opacity Classification System III. Anterior chamber volume (ACV), axial anterior chamber depth (ACD), and central corneal thickness (CCT) were measured using the Pentacam system (Oculus Inc., Wetzlar, Germany). Ocular biometry was performed using the IOL Master system (IOL Master 500, Carl Zeiss Meditec, Jena, Germany), which uses signals from the tear film and retinal pigment epithelium to measure axial length (AL). Corneal endothelial cell number and morphology were examined by CellChek XL (Konan Medical, Irvine, CA, USA). Retinal macular thickness was measured using spectralis OCT volume scans (Heidelberg Engineering, Heidelberg, Germany). Patients underwent slit lamp examination was performed to determine tear breakup time (TBUT) and the Schirmer test. Patients were also administered questionnaires to assess their occupation, average daily activity, and use of vitamin D supplements.

All cataract surgeries were performed by a single experienced surgeon (S.J.K.). After instillation of topical anesthesia, approximately 150–200 μL of aqueous humor was collected according to anterior chamber depth with a 30-gauze tuberculin syringe through the clear cornea near the limbus before making main incision (2.2mm), followed by completion of cataract surgery.

### Laboratory analysis

All aqueous humor samples were taken to the laboratory immediately after collection, and their vitamin D concentrations were measured. By contrast, all serum specimens were stored at − 70 °C until analyzed.

Concentrations of total 25(OH) D were measured using Elecsys Vitamin D Total Kits with the Cobas e602 module (Roche Diagnostics, Mannheim, Germany), an electrochemiluminescence assay that measures two forms of 25-hydroxy vitamin D (D_2_ and D_3_) in three steps. During the first step, bound 25(OH) D is released from the vitamin D binding protein (VDBP). In the second step, the released 25(OH) D binds to added ruthenium labeled VDBP to form a complex. In the third step, the unbound ruthenium labeled VDBP is removed by the addition of streptoavidin-coated microparticles and 25(OH) D is labeled with biotin. Application of voltage to the electrode then induces the ruthenimun labeled VDBP-25(OH) D complex to emit chemiluminescence, which is measured with a photomultiplier. The signals produced were inversely proportional to the 25(OH) D concentrations in the original samples. Measured concentrations are standardized using an instrument-generated calibration curve [30]. The volume of each aqueous humor collected from the anterior chamber was approximately 150–200 μL and the actual volume analyzed by the Elecsys kits was 40 μL.

### Statistical analysis

Categorical data are presented as number (%), and continuous data as median (interquartile range; IQR) because most of the continuous data did not satisfy the normality and homoscedasticity assumptions of the Shapiro-Wilk test and Leven’s F test, respectively. Spearman’s rank correlation analysis was used to assess the correlation between vitamin D concentrations in serum and aqueous humor. The concentrations of vitamin D in aqueous humor and serum were compared in the senile and diabetic cataract groups using the Mann-Whitney U test or the Kruskal-Wallis test.

The proportions of variables in the senile and diabetic cataract groups were compared using Fisher’s exact test. Multivariate logistic regression analysis was performed to calculate the adjusted odds ratio (OR) of diabetic cataract relative to senile cataract. The adjusted variables selected for the multivariate analysis were those with *P* values <0.15 in univariate analysis and without multiple collinearity. In addition, clinically important variables were included in the multivariate analysis for adjustment. The adjusted OR of aqueous humor vitamin D was calculated separately based on quartiles (Model 1) and as a continuous variable (Model 2).

Factors affecting the vitamin D concentrations in aqueous humor and serum were assessed by multivariate linear regression analysis using covariates with *P* values ≤0.15 in univariate analysis as well as covariates related to serum vitamin D concentration. Because the concentrations of vitamin D in aqueous humor and serum did not satisfy the normality assumption, the data were transformed by the fractional rank probability method [31].

All statistical analyses were performed with SAS version 9.4 software (SAS Institute Inc., Cary, NC, USA). A two tailed *P* value <0.05 was considered statistically significant.

## Results

### Subject characteristics

The present study enrolled 136 patients, 87 with senile cataract and 49 with diabetic cataract; their demographic and clinical characteristics are shown in Table 1. Vitamin D concentrations were significantly higher in men than in women, both in aqueous humor (11.5 vs. 7.5 ng/mL, *P* = 0.010) and serum (20.1 vs. 14.2 ng/mL, *P* = 0.02). In addition, vitamin D concentration in aqueous humor was significantly higher in patients with diabetic than with senile cataract (12.6 vs. 8.8 ng/mL, *P* = 0.006). There were no other significant differences in demographic and clinical factors between the two groups (all *P* > 0.05).

**Table 1:** Relationships of demographic and clinical characteristics with vitamin D levels in aqueous humor and serum from patients with cataract

	N	Aqueous humor 25(OH) D level	*P* value	Serum 25(OH) D level	*P* value
		Median (IQR)		Median (IQR)	
Demographic characteristics					
Total number	136	10.3 (5.5–16)		15.6 (11.6–25.8)	
Sex			0.010		0.028
Male	69	11.5 (8.1–17.3)		20.1 (13.4–27.3)	
Female	67	7.5 (4.7–14.4)		14.2 (10.8–23.2)	
Age (yr)			0.571		0.702
≤ 59	36	9.7 (4.6–13.9)		14.6 (9.3–27.9)	
60–69	49	10.0 (4.7–17.4)		19.6 (12.9–23.3)	
70–79	34	10.1 (5.8–14.5)		15.4 (13.0–29.6)	
≥ 80	17	11.0 (8.9–17.9)		13.6 (10.7–27.8)	
Average outdoor activity time (hr/day)			0.146		0.432
< 2	51	11.5 (6.6–16.3)		14.6 (11.1–23.2)	
2–6	61	8.7 (4.7–13.6)		16.1 (12.5–28.4)	
≥ 6	24	13.0 (6.0–17.7)		20.1 (12.0–23.6)	
Occupation			0.373		0.185
None	48	10.8 (7.0–17)		15.2 (11.5–23.8)	
Housewife/clerk	49	8.2 (4.8–13.8)		14.6 (10.8–23.4)	
Farmer/outdoor worker	39	10.5 (6–17.4)		20.1 (12.9–29.6)	
Vitamin D supplementation			0.451		0.144
No	127	10.4 (5.4–16.7)		15.3 (11.6–24.1)	
Yes	9	8.1 (5.8–12.0)		29.2 (15.2–30.1)	
Clinical characteristics					
Type of cataract			0.006		0.631
Senile	87	8.8 (4.4–13.6)		16.1 (11.6–26.3)	
Diabetic	49	12.6 (7.5–20.2)		14.7 (12.4–23.2)	
Hypertension			0.276		0.879
No	65	9.1 (4.7–14.6)		15.6 (11.9–26.3)	
Yes	71	10.9 (5.8–16.7)		15.6 (11.6–24.9)	
Lung disease			0.195		0.306
No	108	9.9 (5.5–15.6)		16.2 (12.1–25.8)	
Yes	27	12.6 (7.4–17.9)		13.5 (10.8–26.8)	
Previous thyroid operation			0.232		0.122
No	134	10.4 (5.5–16.2)		15.7 (11.7–26.0)	
Yes	2	6 (3.0–9.0)		9.5 (5.8–13.2)	

Tables 2 and 3 summarize the proportional differences in each factor according to the type of cataract. Most clinical factors and ophthalmological characteristics, such as sex, age, average daily outdoor activity time, occupation, and use of vitamin D supplements, did not differ significantly between the groups of patients with senile and diabetic cataract (Table 2). The prevalence of hypertension was significantly higher in patients with diabetic than senile cataract (*P* = 0.012), whereas the percentage of patients with AL > 24 mm was significantly higher in the senile cataract than in the diabetic cataract group (*P* = 0.003; Table 3).

**Table 2:** Demographic and clinical characteristics of patients with senile and diabetic cataract

	N (%)	Type of cataract		*P* value
		Senile, N (%)	Diabetic, N (%)	
Demographic characteristics				
Total number	136 (100.0)	87 (64.0)	49 (36.0)	
Sex				0.479
Male	69 (50.7)	42 (60.9)	27 (39.1)	
Female	67 (49.3)	45 (67.2)	22 (32.8)	
Age (yr)				0.310
≤ 59	36 (26.5)	27 (75.0)	9 (25.0)	
60–69	49 (36.0)	28 (57.1)	21 (42.9)	
70–79	34 (25.0)	20 (58.8)	14 (41.2)	
≥ 80	17 (12.5)	12 (70.6)	5 (29.4)	
Average outdoor activity time (hr/day)				0.303
< 2	51 (37.5)	34 (66.7)	17 (33.3)	
2–6	61 (44.9)	41 (67.2)	20 (32.8)	
≥ 6	24 (17.6)	12 (50.0)	12 (50.0)	
Occupation				0.330
None	48 (35.3)	27 (56.3)	21 (43.8)	
Housewife/clerk	49 (36.0)	32 (65.3)	17 (34.7)	
Farmer/outdoor worker	39 (28.7)	28 (71.8)	11 (28.2)	
Vitamin D supplementation				0.722
No	127 (93.4)	82 (64.6)	45 (35.4)	
Yes	9 (6.6.0)	5 (55.6)	4 (44.4)	
Clinical characteristics				
Hypertension				0.012
No	65 (47.8)	49 (75.4)	16 (24.6)	
Yes	71 (52.2)	38 (53.5)	33 (46.5)	
Lung disease				0.506
No	108 (79.4)	67 (62.0)	41 (38.0)	
Yes	27 (19.9)	19 (70.4)	8 (29.6)	
Previous thyroid operation				1.000
No	134 (98.5)	86 (64.2)	48 (35.8)	
Yes	2 (1.5)	1 (50.0)	1 (50.0)

**Table 3:** Ophthalmologic characteristics and 25(OH) concentrations in patients with senile and diabetic cataract

	N (%)	Type of cataract		*P* value
		Senile, N (%)	Diabetic, N (%)	
Ophthalmologic examination results				
BCVA (Snellen)				1.000
≤ 20/125	14 (10.3)	9 (64.3)	5 (35.7)	
20/100–20/40	61 (44.9)	38 (62.3)	23 (37.7)	
≥ 20/32	41 (30.1)	25 (61.0)	16 (39.0)	
IOP (mmHg)				1.000
≤ 20	131 (96.3)	84 (64.1)	47 (35.9)	
> 20	5 (3.7)	3 (60.0)	2 (40.0)	
NC				0.239
0–1	12 (8.8)	9 (75.0)	3 (25.0)	
2–3	99 (72.8)	59 (59.6)	40 (40.4)	
4–5	25 (18.4)	19 (76.0)	6 (24.0)	
NO				0.135
0–1	6 (4.4)	5 (83.3)	1 (16.7)	
2–3	106 (77.9)	63 (59.4)	43 (40.6)	
4–5	24 (17.6)	19 (79.2)	5 (20.8)	
PSC				0.054
0–1	94 (69.1)	66 (70.2)	28 (29.8)	
2–3	37 (27.2)	18 (48.7)	19 (51.4)	
4–5	5 (3.7)	3 (60.0)	2 (40.0)	
Central macular thickness (μm)				0.534
≤ 250	28 (20.6)	18 (64.3)	10 (35.7)	
250–300	69 (50.7)	41 (59.4)	28 (40.6)	
> 300	28 (20.6)	20 (71.4)	8 (28.6)	
Schirmer (mm)				0.395
≤ 10	33 (24.3)	17 (51.5)	16 (48.5)	
> 10	70 (51.5)	43 (61.4)	27 (38.6)	
Spherical equivalent (D)				0.186
≤ −3.0	34 (25)	26 (76.5)	8 (23.5)	
-3.0–0.0	53 (39)	29 (54.7)	24 (45.3)	
0.0–3.0	32 (23.5)	20 (62.5)	12 (37.5)	
> 3.0	1 (0.7)	1 (100.0)	0 (0.0)	
Central corneal thickness (μm)				0.675
≤ 520	31 (22.8)	21 (67.7)	10 (32.3)	
> 520	105 (77.2)	66 (62.9)	39 (37.1)	
ECC (cells/mm^2^)				0.468
≤ 1000	3 (2.2)	3 (100.0)	0 (0.0)	
1000–2000	12 (8.8)	7 (58.3)	5 (41.7)	
> 2000	114 (83.8)	71 (62.3)	43 (37.7)	
Axial length (mm)				0.003
20–24	95 (69.9)	53 (55.8)	42 (44.2)	
≥ 24	41 (30.1)	34 (82.9)	7 (17.1)	
Anterior chamber depth (mm)				0.203
≤ 2	11 (8.1)	5 (45.5)	6 (54.6)	
2–3	93 (68.4)	58 (62.4)	35 (37.6)	
> 3	32 (23.5)	24 (75.0)	8 (25.0)	
Anterior chamber volume (mm^3^)				0.368
≤ 100	33 (24.3)	19 (57.6)	14 (42.4)	
100–150	69 (50.7)	43 (62.3)	26 (37.7)	
> 150	34 (25.0)	25 (73.5)	9 (26.5)	
25(OH) D concentrations (in quartiles, ng/mL)				
Serum				0.686
Q1 (≤11.78)	35 (25.7)	23 (65.7)	12 (34.3)	
Q2 (11.78–16.04)	36 (26.5)	20 (55.6)	16 (44.4)	
Q3 (16.04–25.81)	31 (22.8)	21 (67.7)	10 (32.3)	
Q4 (>25.81)	34 (25.0)	23 (67.7)	11 (32.4)	
Aqueous humor				0.034
Q1 (≤5.5)	35 (25.7)	27 (77.1)	8 (22.9)	
Q2 (5.5–10.43)	35 (25.7)	21 (60.0)	14 (40.0)	
Q3 (10.43–16.16)	33 (24.3)	24 (72.7)	9 (27.3)	
Q4 (>16.16)	33 (24.3)	15 (45.5)	18 (54.6)	

Abbreviations: *25(OH) D* 25-hydroxyvitamin D, *BCVA* Best-corrected visual acuity, *IOP* Intraocular pressure, *NC* Nuclear color, *NO* Nuclear opalescence, *PSC* Posterior subcapsular, *D* Diopters, *ECC* Endothelial cell count

### Total 25(OH) concentrations in aqueous humor and serum

**Fig. 1:**
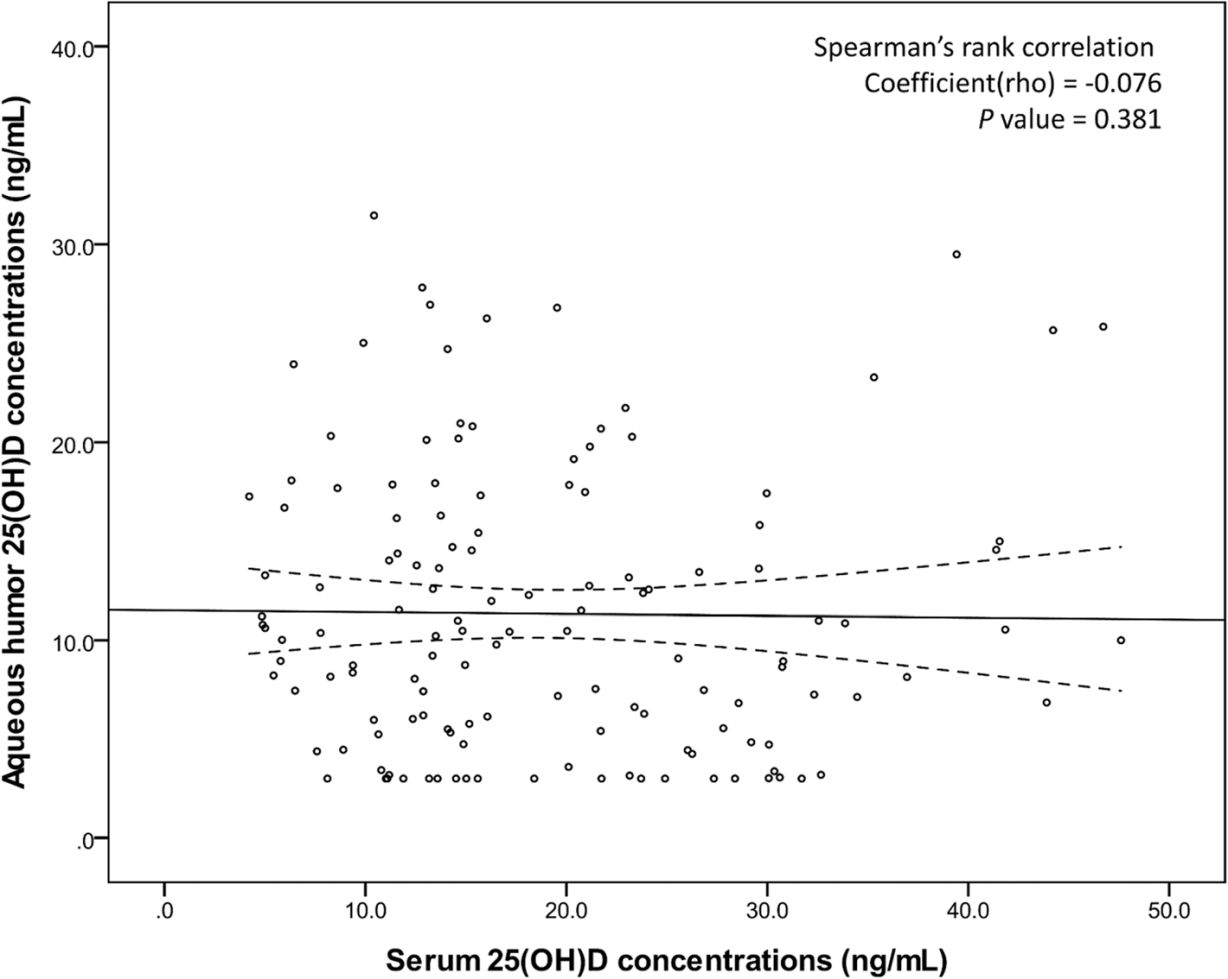
Correlation between 25(OH) D concentrations in serum and aqueous humor

The median (IQR) 25(OH) D concentration was 10.3 (5.5–16) ng/mL in aqueous humor and 15.6 (11.6–25.8) ng/mL in serum (Table 1). Measurements using the Cobas e602 module were possible if the sample volume was > 400 μL (data not shown). 25(OH) D concentrations in aqueous humor and serum did not show significant correlation (*P* = 0.381; Fig. 1). Univariate analysis showed that sex and type of cataract were factors related to 25(OH) D concentration in aqueous humor and that sex was the only factor related to serum 25(OH) D concentration (Table 1). The median concentration was 4.0 ng/mL lower in women than in men (*P* = 0.010) and 3.8 ng/mL lower in patients with senile than diabetic cataract (*P* = 0.006). Median (IQR) of aqueous humor vitamin D levels according to nuclear color (NC) 0–1, 2–3 and 4–5 stages were 12.8(8.8–14.3), 10.4(5.8–16.3) and 8.2(4.4–16.2) (*P* = 0.447), respectively. And the median (IQR) of aqueous humor vitamin D levels according to nuclear opalescence (NO) 0–1, 2–3 and 4–5 stages were 12.9(4.7–14.0), 10.4(5.8–16.3) and 8.4(4.5–15.3) (*P* = 0.702), respectively (data not shown).

### Comparison of ophthalmologic examination results and 25(OH) concentrations in aqueous humor and serum according to the type of cataract

**Fig. 2:**
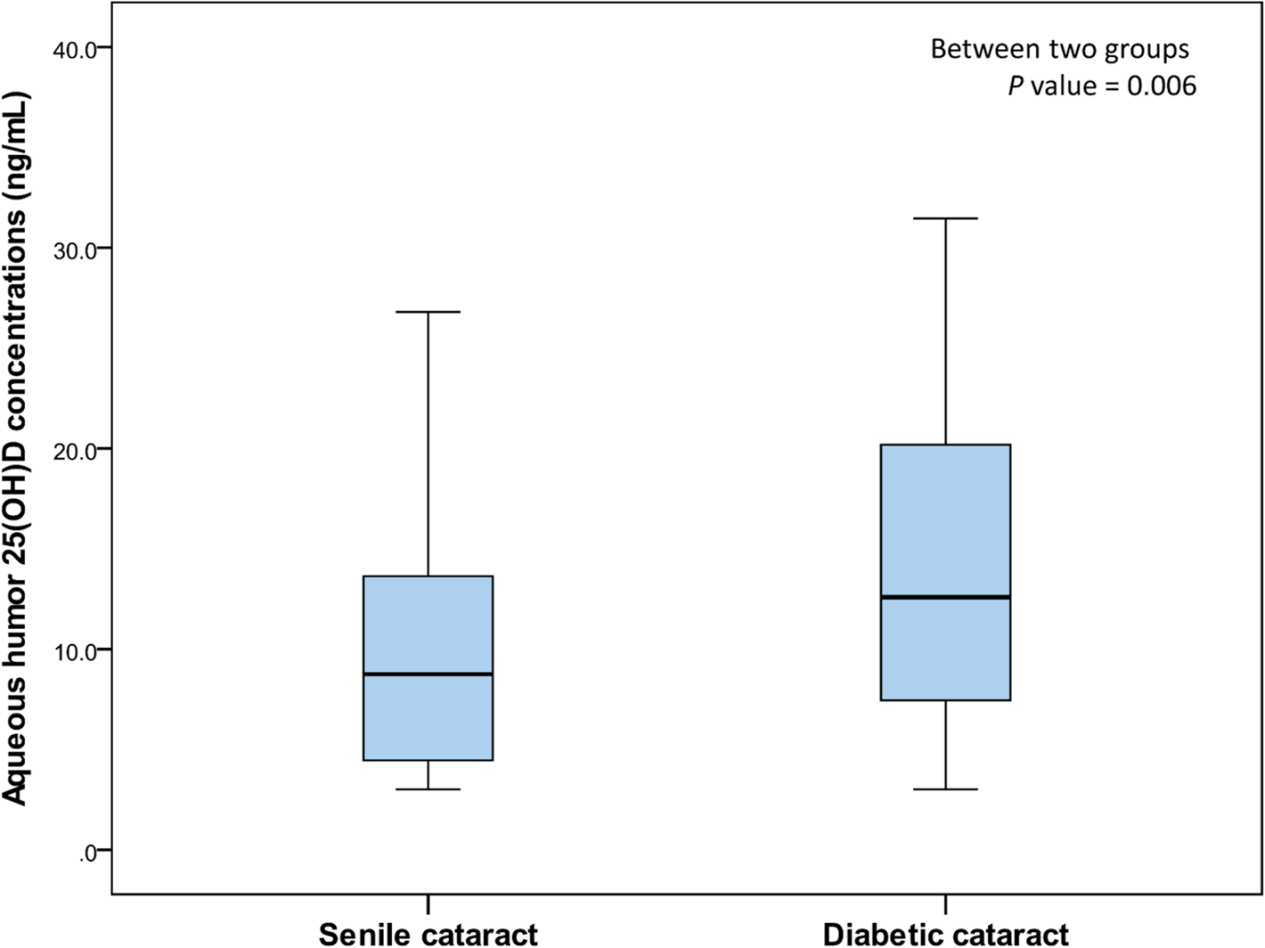
Aqueous humor 25(OH) D concentrations in patients with senile and diabetic cataract

Ophthalmologic examination results and total 25(OH) concentrations in aqueous humor and serum were also compared in groups of patients with diabetic and senile cataract (Table 3). None of the ophthalmologic examination results differed significantly in these two groups (all *P* > 0.05). When 25(OH) D concentrations in aqueous humor and serum were analyzed by quartile, diabetic cataract showed the highest proportion (54.6%) in the fourth quartile (Q4), with the highest concentration of 25(OH) D in aqueous humor (*P* = 0.034). 25(OH) D concentrations in aqueous humor differed significantly in the groups of patients with senile and diabetic cataract (*P* = 0.006; Fig. 2).

### Multivariate logistic regression analysis of factors associated with diabetic cataract

Factors included in the multivariate logistic regression analysis were 25(OH) D concentration in aqueous humor, hypertension, and AL, all of which were significant in univariate analysis, and sex, age, and average daily outdoor activity time, which were considered clinically important. When 25(OH) D concentrations in aqueous humor were analyzed by quartile, diabetic cataract was significantly more frequent in Q4 than in Q1 subjects (Model 1: adjusted OR = 4.36; 95% confidence interval [CI] = 1.33–14.34; *P* = 0.015), and diabetic cataract was significantly less frequent in subjects with AL ≥24 mm than <24 mm (Model 1: adjusted OR = 0.24; 95% CI = 0.08–0.70; *P* = 0.009). In Model 2, in which 25(OH) D concentrations were analyzed as a continuous variable, a 5 ng/mL increase in 25(OH) D level in aqueous humor increased the OR for diabetic cataract by 1.2 (95% CI = 0.38–1.91; Table 4). No other factor was associated with diabetic cataract.

**Table 4:** Associations between demographic and clinical characteristics and the likelihood of diabetic cataract after adjusting for confounders

Variables	Model 1			Model 2		
	Adjusted OR	95% CI	*P* value	Adjusted OR	95% CI	*P* value
Aqueous humor 25(OH) D level (per 5 ng/mL increase)				1.20	1.00–1.30	0.005
Aqueous humor 25(OH) D Q1 (vs. ≤5.5 ng/mL)						
5.5 ng/mL < Q2 ≤ 10.43 ng/mL	2.18	(0.69–6.90)	0.185			
43 ng/mL < Q3 ≤ 16.16 ng/mL	1.08	(0.30–3.85)	0.902			
16.16 ng/mL < Q4	4.36	(1.33–14.34)	0.015			
Sex						
Female (vs. male)	0.69	(0.30–1.60)	0.389	0.85	(0.38–1.91)	0.691
Age						
60–69 yr (vs. ≤59 yr)	1.74	(0.59–5.13)	0.314	1.92	(0.66–5.54)	0.230
70–79 yr (vs. ≤59 yr)	1.19	(0.38–3.79)	0.764	1.13	(0.36–3.60)	0.833
≥ 80 yr (vs. ≤59 yr)	0.59	(0.13–2.63)	0.490	0.64	(0.15–2.85)	0.562
Average outdoor activity time, hr./day						
2–6 (vs. <2)	0.85	(0.34–2.11)	0.729	0.97	(0.40–2.36)	0.948
> 6 (vs. <2)	1.39	(0.46–4.26)	0.560	1.65	(0.54–4.99)	0.378
Hypertension						
Yes (vs. no)	2.27	(0.98–5.28)	0.056	1.97	(0.87–4.47)	0.103
Axial length						
≥ 24 mm (vs. <24 mm)	0.24	(0.08–0.70)	0.009	0.22	(0.07–0.63)	0.005

Hosmer & Lemeshow model fit: Model 1, chi-square = 5.42, *P* = 0.712; Model 2, chi-square = 2.78, *P* = 0.947

25(OH) D, 25-hydroxyvitamin D; HTN, hypertension

### Multivariate linear regression analysis for determining factors affecting aqueous humor and serum 25 (OH) D

The factors affecting 25(OH) D level in aqueous humor were sex and type of cataract. 25(OH) D concentrations in aqueous humor were 2.79 ng/mL higher (95% CI = 0.53–5.05 ng/mL, *P* = 0.016) in men than in women and 2.68 ng/mL (95% CI = 0.34–5.01 ng/mL, *P* = 0.025) higher in patients with diabetic than senile cataract (Table 5). In addition, serum vitamin D concentration was 7.8 ng/mL (95% CI = 0.34–5.01, *P* = 0.025) higher in patients who did than did not take vitamin D supplements (Table 6).

**Table 5:** Multivariate linear regression analysis of factors associated with 25(OH)D concentrations in aqueous humor

	Anterior chamber 25(OH)D concentration		
	Regression coefficient	95% CI	*P* value
Sex			
Female vs (ref. male)	-2.79	(-5.05 – -0.53)	0.016
Type of cataract			
Diabetic cataract vs (ref. senile)	2.68	(0.34–5.01)	0.025
Average outdoor activity time (hr/day)			
2–6 (ref. <2)	-2.04	(-4.49–0.42)	0.103
>6 (ref. <2)	-0.32	(-3.54–2.89)	0.843
ECC (cells/mm^2^)			
1000–2000 vs (ref. <1000)	7.47	(-0.68–15.63)	0.072
>2000 vs (ref. <1000)	3.3	(-4.16–10.76)	0.383

*ECC* endothelial cell count

**Table 6:** Multivariate linear regression analysis of factors associated with 25(OH)D concentrations in serum

	Serum 25(OH)D concentration		
	Regression coefficient	95% CI	*P* value
Sex			
Female vs (ref. male)	-3.35	(-6.85–0.15)	0.060
Vitamin D supplementation			
Yes vs (ref. no)	7.8	(0.37–15.22)	0.040
Average outdoor activity time (hr/day)			
2–6 (ref. <2)	1.78	(-1.96–5.53)	0.347
>6 (ref. <2)	0.56	(-4.28–5.41)	0.818
Previous thyroid operation			
Yes vs (ref. no)	-10.89	(-29.89–8.1)	0.258
NC			
2–3 (ref. 0–1)	3.42	(-2.89–9.72)	0.286
4–5 (ref. 0–1)	-2.96	(-10.32–4.41)	0.428
ECC (cells/mm^2^)			
1000–2000 vs (ref. <1000)	-11.75	(-39.63–16.13)	0.405
>2000 vs (ref. <1000)	-11.13	(-38.61–16.35)	0.424
Central macular thickness			
250–300 vs (ref. ≤250)	1.48	(-3.03–5.98)	0.516
>300 vs (ref. ≤250)	2.41	(-2.68–7.49)	0.351
Axial length			
>24 mm vs (ref. 20–24 mm)	-2.41	(-6.52–1.71)	0.250

*25(OH)D* 25-hydroxyvitamin D, *NC* nuclear color, *ECC* endothelial cell count

## Discussion

Serum 25(OH) D status has been associated with ocular health and disease [32], including with ocular surface diseases such as allergic conjunctivitis and dry eye [15, 33, 34]. Fewer studies, however, have investigated the association between ocular diseases and 25(OH) D in tear fluid. Furthermore, to our knowledge, no study to date has measured the concentration range of 25(OH) D in human aqueous humor or the association of 25(OH) D with ocular diseases such as cataract. The present study was therefore designed to determine whether 25(OH) D is present at measurable concentrations in human aqueous humor, whether 25(OH) D concentrations in aqueous humor and serum correlated, and whether 25(OH) D concentrations in aqueous humor were associated with cataract. We confirmed that 25(OH) D was present in human aqueous humor at a concentration directly measurable by an electrochemiluminescent assay used clinically to measure 25 (OH) D. However, the concentration of 25(OH) D in aqueous humor did not correlate with its concentration in serum. Interestingly, the present study found that 25(OH) D level was significantly higher in aqueous humor of patients with diabetic than with senile cataract.

The present study found no correlation between vitamin D concentrations in aqueous humor and serum of patients with cataract. Multivariate linear regression analysis showed that sex and type of cataract were factors affecting vitamin D concentration in aqueous humor. Specifically, 25(OH) D concentrations in aqueous humor were 2.79 ng/mL higher (95% CI = 0.53–5.05 ng/mL, *P* = 0.016) in men than in women and 2.68 ng/ml (95% CI = 0.34–5.01 ng/mL, *P* = 0.025) higher in patients with diabetic than senile cataract. Serum 25(OH) D levels were also significantly higher in men than in women and lower in patients with severe than less severe nuclear cataract. The mean baseline serum vitamin D level in this study (15.6 ng/mL; range, 11.6–25.8 ng/mL) was lower than in previous studies (20–110 nmol/L), although our finding that serum vitamin D levels were higher in men than in women was consistent with previous results [35]. Earlier studies also suggested that lower serum 25(OH) D levels were associated with a higher risk of nuclear cataract and significantly associated with an elevated risk of glaucoma in women [35, 36]. Furthermore, lower serum 25(OH) D concentration was associated with longer AL and a higher risk of myopia, resulting in a significantly higher prevalence of myopia in individuals with vitamin D deficiency than in individuals with sufficient vitamin D levels [35, 36].

We found that total 25(OH) concentrations in aqueous humor and serum were not significantly correlated in cataract patients, suggesting that vitamin D in aqueous humor may not be affected by serum vitamin D concentration and that serum vitamin D concentrations may not be associated with intraocular diseases such as cataract. In addition, these findings could lead to additional studies determining whether vitamin D crosses the blood-aqueous humor barrier via a special transport system. Most (85–90%) of the circulating 25(OH) D in serum is tightly bound to the 58 kDa sized vitamin D-binding protein (VDBP), with a smaller percentage (10–15%) loosely bound to albumin, and less than 1% circulating in free unbound form [37–39]. VDBP-bound 25(OH) D may be transported to tear fluid by the megalin/cubilin transport system [40], which has been detected in animal lacrimal and accessory glands [18]. A study of 48 healthy subjects reported a correlation between 25(OH) D levels in serum and tear fluid, suggesting that serum vitamin D was transferred to tear fluid by the megalin/cubilin system present in lacrimal and accessory glands. Our results suggest, however, that the mechanisms involved in the synthesis and migration of vitamin D differ in aqueous humor and tear fluid. Investigation of this hypothesis requires further studies to determine whether megalin/cubilin is present in the eye, other than in the lacrimal and accessory glands. In addition, because the eye is exposed to the sun, it can produce or activate vitamin D [32], suggesting that the production of vitamin D in intraocular tissues containing lens epithelial cells and/or lens protein may explain the lack of correlation between vitamin D concentrations in aqueous humor and serum.

We found that the concentration of vitamin D in aqueous humor was significantly higher in patients with diabetic than senile cataract, a result confirmed by multivariate logistic regression analysis and multivariate linear regression analysis. Although this study was the first to measure vitamin D concentration in aqueous humor, a large-scale study involving 16,000 patients reported that serum 25(OH) D levels were inversely associated with the risk of nuclear cataract. We found that serum 25(OH) D concentrations did not differ significantly in patients with senile and diabetic cataract (*P* < 0.686), whereas 25(OH) D concentration in aqueous humor was significantly higher in patients with diabetic than senile cataract. To explain this finding, we focused on the anti-cataractogenic role of vitamin D, and the increases in inflammatory responses and oxidative stress in patients with diabetes. Vitamin D is reported to regulate the immune system and to suppress oxidative stress [41, 42]. Vitamin D exerts an anti-inflammatory effect by decreasing the proliferation of lymphocytes and natural killer cells and the secretion of several proinflammatory cytokines [41]. Additionally, calcitriol, the active metabolite of vitamin D, was shown to act as a potent anti-oxidant [42]. In patients with diabetes, inflammatory responses are up regulated [43] and oxidative stress is much higher than normal [44], resulting in vascular and other complications. Vitamin D suppresses oxidative stress, which induces cataract [45, 46]. Thus, inflammatory responses and oxidative stress were likely greater in the patients with diabetic than senile cataract, with the former showing greater increases in 25(OH) D levels in aqueous humor. The effect of diabetes on the blood-aqueous barrier [47] may explain the reason that vitamin D levels in the aqueous humor were higher in patients with diabetic than senile cataract. However, the reason that serum 25(OH) D concentrations did not differ in the two groups remains unclear; further studies are therefore required.

This study had several limitations. First, the number of patients was relatively small. Second, because most patients with diabetic cataract were elderly, we could not completely exclude the possibility that age was a confounding factor in the development of cataract. Third, we did not exclude the effects of insulin and various hypoglycemic drugs that could affect vitamin D concentrations in serum or aqueous humor. Fourth, we could not compare our patients with a control group of patients without cataracts, because, for ethical reasons, we could not obtain aqueous humor from the latter. To elucidate the role of vitamin D in aqueous humor, a larger clinical study, including a control group of patients without cataracts, is required. Finally, total 25(OH) D levels in this study were not measured by LC-MS/MS, the method considered the gold standard for measuring 25(OH) D concentrations. However, the assay used in this study, the Elecsys Vitamin D Total Kit with the Cobas e602 module (Roche Diagnostics), is reported to yield results comparable to those of the LC-MS/MS method [48].

## Conclusions

In conclusion, this study is the first to show that vitamin D is present in aqueous humor and to report its concentrations in groups of patients with diabetic and senile cataract. We found that 25(OH) concentrations in aqueous humor and serum did not correlate with each other, whereas 25(OH) D concentrations in aqueous humor were higher in patients with diabetic than senile cataract. These findings suggest that studies of the effects of vitamin D in the eye should include measurements of vitamin D levels in aqueous humor.

## Data Availability

The data of the current study are available from the corresponding author on reasonable request.

## Abbreviations

1α, 25 (OH) D: 1α, 25-dihydroxyvitamin D
25 (OH) D: 25-hydroxyvitamin D
ACD: Anterior chamber depth
ACV: Anterior chamber volume
AL: Axial length
CCT: Central corneal thickness
CI: Confidence interval
HPLC: High performance liquid chromatography
IQR: Interquartile range
LC-MS/MS: Liquid chromatography with tandem mass spectrometry
OR: Odds ratio
SVCT2: Sodium-dependent vitamin C transporter 2
TBUT: Tear breakup time
VDBP: Vitamin D-binding protein
